# 16. An Ambulatory Quality Improvement Initiative to Optimize Influenza Vaccination Amongst Adults Living with HIV During the COVID-19 Pandemic

**DOI:** 10.1093/ofid/ofab466.218

**Published:** 2021-12-04

**Authors:** Deborah A Kahal, Christopher James, Brian Wharton, Sherine Eaddy, Elizabeth Gaines, Karen Henry, Luis Juarez, Bincsik K Arlene

**Affiliations:** 1 Christiana Care Health System, Media, Pennsylvania; 2 ChristianaCare, Wilmington, Delaware; 3 ChristitanaCare, Wilmington, Delaware

## Abstract

**Background:**

Seasonal influenza vaccination decreases individual and population-level morbidity and mortality, mitigates risk of acquiring influenza-like illness, and prevents healthcare system overburdening. Vaccination is important for people living with HIV (PLWH) who have increased risk for severe disease, hospitalization, and poor outcomes. Moreover, influenza vaccination has been associated with decreased COVID-19 mortality in older patients. Historical annual adult influenza vaccinations rates at the study site were 65%, exceeding local and national benchmarks. Amidst COVID-19, we recognized a need to increase influenza vaccination rates.

**Methods:**

A multifaceted, bundled quality improvement (QI) initiative aimed to achieve ≥ 80% influenza vaccination coverage for the 2020-21 season in PLWH ≥18 years of age at our Wilmington site (N=750). Stakeholders were identified, and a voluntary multidisciplinary team formed to lead the initiative (Fig. 1). Fishbone diagram outlined clear, rapidly implementable, and reproducible levers for change (Fig. 2). Physical and virtual space changes included: diverse clinical displays (visuals, patient materials), phone messaging, and virtual platform use. Staff education and updates were consistently provided by the team. Institutional Review Board exemption was received, and electronic medical record and CareWare data were extracted from 1 Oct 2020 through 31 March 2021. All external vaccinations were confirmed. Overall and eligible in-clinic vaccination rates were updated and displayed weekly.

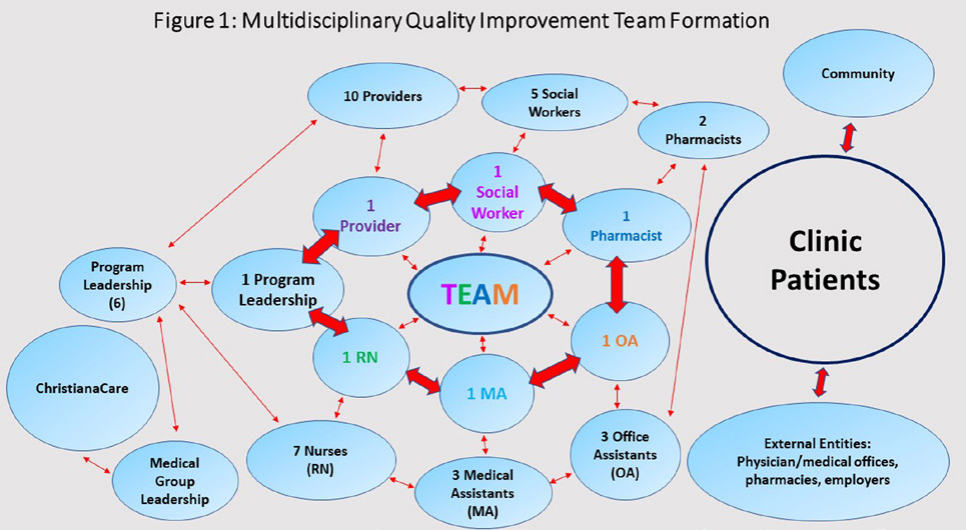

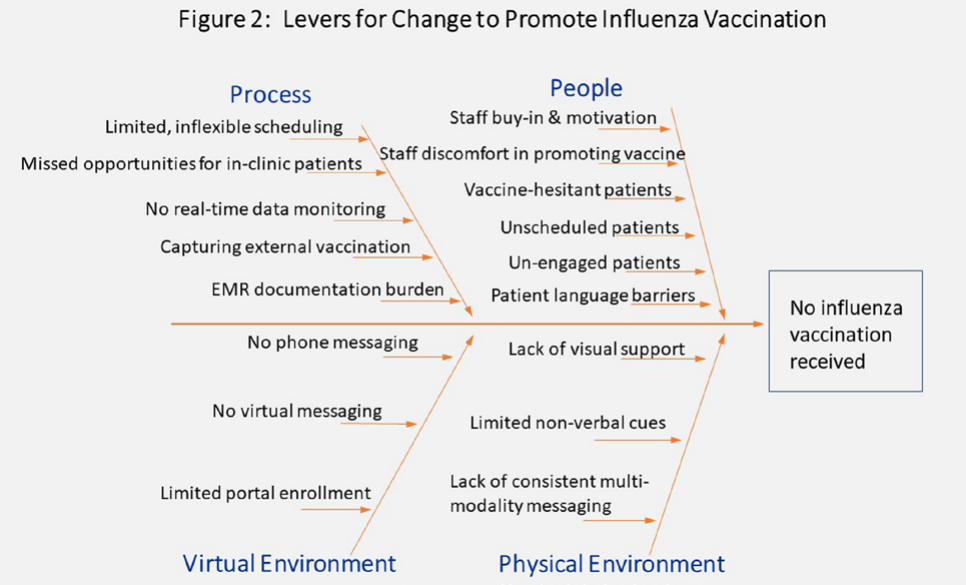

**Results:**

86% vaccination coverage was achieved (Fig. 3) with a median weekly in-clinic vaccination rate of 67% (Fig. 4).

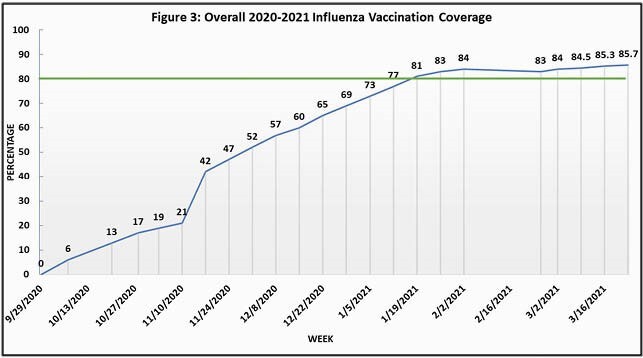

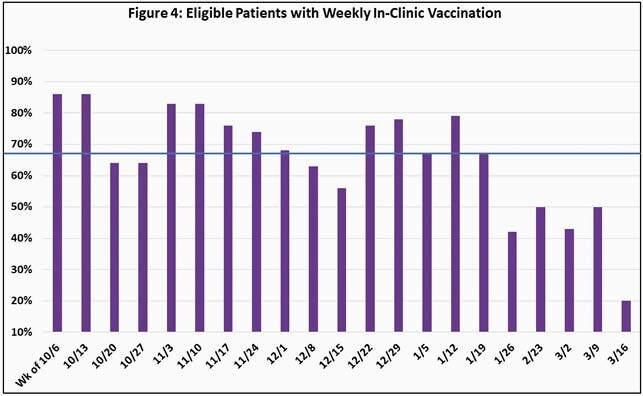

**Conclusion:**

A QI project to improve 2020-21 influenza vaccination rates exceeded our goal in adult PLWH at an urban mid-Atlantic HIV clinic during the COVID-19 pandemic. A multidisciplinary approach that engaged stakeholders was vital to success. Rapid roll-out of changes was challenging, requiring flexibility and clear communication. Data collection was consistent, albeit imperfect, and needs enhancement. Elucidating the effects of each change and the COVID-19 pandemic on vaccination rates is not yet known. Lessons learned may be applicable to other ambulatory settings and will inform future vaccination efforts.

**Disclosures:**

**Deborah A. Kahal, MD,MPH, FACP**, **Gilead** (Speaker’s Bureau)**Viiv** (Speaker’s Bureau)

